# Oxidative Stress in Skeletal Muscle

**DOI:** 10.3390/antiox11071299

**Published:** 2022-06-29

**Authors:** Giorgio Fanò-Illic, Stefania Fulle

**Affiliations:** 1 IIM-Interuniversity Institute of Myology, University “G. d’Annunzio” of Chieti-Pescara, 66100 Chieti, Italy; fanoillic@gmail.com; 2 Campus of Free University of Alcatraz, Santa Cristina di Gubbio, 06024 Gubbio, Italy; 3 Department of Neuroscience Imaging and Clinical Sciences, University “G. d’Annunzio” of Chieti-Pescara, 66100 Chieti, Italy

The accumulation of ROS, mainly due to increased mitochondrial production and/or decreased scavenger systems, is often associated with the term oxidative stress, used to define a condition judged to be problematic for muscle cells. However, this situation, which is very evident but transitory during physical activity and of significant intensity and persistence with advancing age, does not only cause detrimental effects on the ability of the muscle fiber to perform work. This is particularly evident both in the ryanodine channels of the sarcoplasmic reticulum and in the mitochondria. In these sites, adequate levels of ROS, particularly H_2_O_2_, can positively modulate organelle activity. This can also occur in synergy with other intracellular messengers such as Zn^2+^ and Ca^2+^ as proposed in the review by Di Filippo et al. [1], in which it is suggested that these response generators (H_2_O_2_, Zn^2+^, Ca^2+^) can interpret environmental conditions by modulating, in the mitochondrion and outside it, their interspecific cooperation. The resulting response is not always the same but depends on the functional state of the muscle cell. Indeed, the altering of H_2_O_2_ levels, in different ways and in different compartments, changes the stable state from eustress to distress (Figure 1).

Additionally, considering this assumption, it is essential to control the level that free radicals reach in the various compartments of the muscle fiber in order to interpret their intracellular activity.

One way to regulate the level of free radicals (especially oxygen-derived ones) in the cell is to use integrators or drugs that can influence the concentration of ROS either by regulating scavenger systems or by acting on their synthesis.

The review by Devrim-Lanpir et al. [2] focuses on the induced effects of using N-acetylcysteine (NAC) due to its precursor role in the production of hepatic glutathione, a natural antioxidant that can significantly modulate exercise-induced oxidative stress. In the various studies considered by the authors, a possible therapeutic role for NAC in oxidative metabolism and the adaptive response to exercise is not always clear-cut. The conflicting data can be interpreted less problematically in light of hormesis theory, especially if it refers to some compartments of the muscle cell such as the mitochondrion and the sarcomeric area.

EJ Calabrese [3] is one of the strongest proponents of this idea: “Hormesis is a biphasic dose–response with specific quantitative features for the amplitude and width of the stimulation. It is highly generalizable and independent of biological model, endpoint, inducing agent, level of biological organization, and mechanism. Hormesis may be induced via direct stimulation or by overcompensation to a disruption of homeostasis. The induction of hormesis by low-level stressor agents not only rapidly upregulates adaptive processes to repair the damage but also protects the adapted system from damage due to a subsequent challenging dose (toxic) within a definable temporal window”.

Another effect induced by dietary supplements is described in the paper by Dieter Blottner et al. [4] who, as part of a long-term bed-rest project (BR) to study muscular atrophy in humans (Toulouse Cocktail Study), used a diet enriched with polyphenols, omega-3, vitamin E, and selenium (Toulouse Cocktail) to assess its effectiveness in the antioxidant defense response and to improve the functional status of muscle.

In this condition, the increased oxidative stress by ROS and reactive nitrogen species (RNS) is evident. On the other hand, the use of the Toulouse Cocktail as a supplement induces changes in the molecular and functional aspects of the muscles analyzed. In particular, glycolysis, the tricarboxylic acid cycle, oxidative phosphorylation, fatty acid beta-oxidation, and mitochondrial transmembrane transport and protein folding were normalized in treated subjects. In addition, the increase in nitrosative redox homeostasis and muscle deterioration under BR conditions were reduced, while the decrease in the antioxidant defense response to nitrosative stress was attenuated by the treatment, suggesting positive effects of the nutritional intervention protocol. 

In the paper published by Lydia de Salazar and co-workers [5] with a parallel randomized double-blind study of different doses of Docosahexaenoic acid (DHA) in male recreational cyclists, the antioxidant capacity of DHA was quantified as a reduction in urinary 8-OHdG levels. The authors thus demonstrated that DHA supplementation has a dose-dependent endogenous antioxidant property during moderate-intensity, long-term aerobic exercise. Supplementing the daily diet with an effective dose of DHA also appears to reduce the exercise-induced oxidative stress generated during long-duration aerobic exercise. 

Different mechanisms can be interconverted according to cellular needs and can work synergistically to modulate and, where appropriate, protect cells from the accumulation of oxidative damage. Unfortunately, this homeostatic system tends to become less efficient with age, first in males and then in females, and ROS accumulates in the tissues. This scenario could represent what happens in elderly muscle as a consequence of the altered functioning of the respiratory chain, reduced fiber regenerative potential, and cellular antioxidant defenses resulting in a state known as sarcopenia. 

Obesity and the resulting insulin resistance accelerate age-related sarcopenia. In this situation, the concentration of lipocalin-2 (LCN2), an iron-binding protein associated with skeletal muscle regeneration, increases. Eun Bee Choi et al. [6], in the paper published in this Special Issue, show that elevated levels of LCN2 in skeletal muscle in leptin-deficient mice are linked to inflammation and oxidative stress. This is caused by the increased expression of the LCN2 gene in the muscle of sarcopenic mice, which therefore functions as a potent pro-inflammatory factor.

Muscle atrophy is related to sarcopenia, and dexamethasone (DEX) promotes proteolysis, which causes muscle atrophy. Based on this assumption, Shintae Kim and co-workers [7] evaluated the effect of aqueous extract of Curcuma longa L. (CLW) on DEX-induced muscle atrophy in mice. The authors observed that after DEX injection, the expression levels of myostatin, MuRF-1, and Atrogin-1 increased. However, these expression levels decreased in the group that was injected with CLW, leading to the conclusion that CLW inhibits muscle atrophy (Figure 2). These results, although preliminary and limited to mice, suggest that CLW could serve as a natural product for the prevention of muscle atrophy by modulating genes linked to muscle atrophy and increasing antioxidant potential.

As part of the debate on the use of drugs to reduce the negative effects of free radical dysregulation in the cell, Ya-Jyun Liang and collaborators [8] propose in their paper a method for synthesizing a polysaccharide derived from Bletilla striata, an earth orchid, known for its antioxidant action. In this study, the authors used a polysaccharide derived from this plant (BSP) combined with a hydroxyapatite carrier (BSP-HAP) as a vehicle for the controlled release of the active ingredient. In this research, BSP-HAP was synthesized by a modified low-temperature co-precipitation process that would be advantageous for the release of BSP. Experiments in animals showed that rats treated with BSP-HAP recovered both endurance and muscle strength. These results are consistent with the idea of a potential application of the BSP-HAP system in the treatment and prevention of sarcopenia in the near future.

On the basis of what has been described so far, sarcopenia can be addressed not only by dietary supplementation but also by changing one’s lifestyle by increasing the type and amount of daily physical activity.

A contribution concerning this possibility comes from the paper by Monica Frinchi et al. [9] in which the dystrophic mouse model is taken as a model of atrophic muscle. Duchenne muscular dystrophy is a lethal progressive disorder, resulting in mechanical uncoupling of the myofibers, oxidative stress, inflammation, etc. As there is currently no valid therapy for its treatment, physical exercise may represent a valid non-invasive therapeutic approach to slow down the progression of the pathology. An analysis of the literature shows that the mdx mouse is the most widely used model for research in this field and that low-intensity aerobic exercise is probably the most suitable form of exercise for achieving positive effects on the trophic state of the muscles (Figure 3). The benefits arise from reduced levels of free radicals and inflammation, leading to improved muscle function by stimulating muscle regeneration and slowing down proteolysis. This would also counteract the effects of sarcopenia when applied to the senescent muscle.

In conclusion, it seems clear that cellular levels (and their compartmentalization) of free radicals should not be regarded merely as potential toxic products for life.

ROS and other radical agents underlie crucial cellular processes by also acting as messengers that can regulate intrinsic signaling pathways through direct and/or crosstalk mechanisms.

Especially in higher organisms, many compartments, such as the nervous system or skeletal and cardiac muscles, are particularly susceptible to changes in redox status, developing a defensive or adaptive response depending on the concentration, source, and duration of pro-oxidative stimuli.

## Figures and Tables

**Figure 1:**
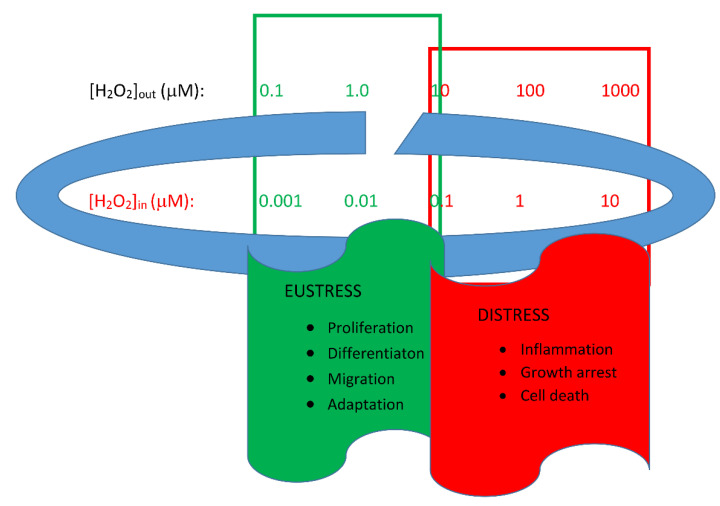
Homeodynamic space. The interpretative model of the regulation of the cellular redox state is based on the existence of a physiological range called the “homeodynamic space”. The relative concentration of H_2_O_2_ can determine situations of eustress and distress. In the situation referred to as eustress, the intracellular H_2_O_2_ concentration is estimated to be 1–10 nM, while the cytosolic concentration is even lower (around 100 pM). The mitochondrial matrix has a peroxide concentration slightly higher than the cytosolic one (up to 20 nM), thus ensuring a gradient between the organelle and the cytoplasm. In the extracellular space, the H_2_O_2_ concentration is between 1 and 5 μM, thus ensuring a gradient of approximately 500 times between the outside and inside of the cell. From Di Filippo et al. [1].

**Figure 2:**
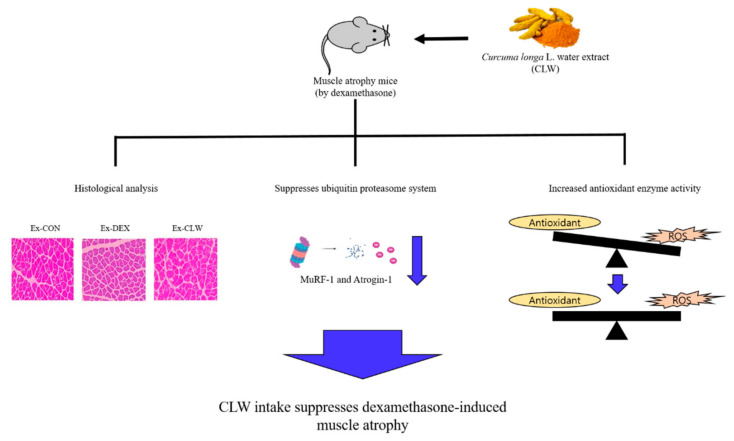
Graphical illustration of how Curcuma longa L. water extract improves dexamethasone-induced sarcopenia by modulating the muscle-related gene and oxidative stress in mice. From Kim, S. et al. [7].

**Figure 3:**
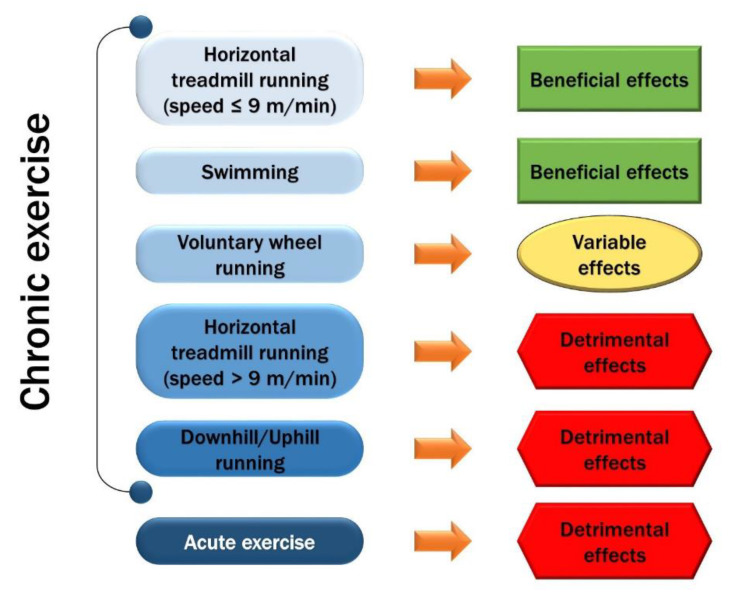
Graphical Abstract. From Frinchi, M. et al. [9].
